# Supplementary figures and images for: One-Step Soft Agar Enrichment and Isolation of Human Lung Bacteria Inhibiting the Germination of Aspergillus fumigatus Conidia
Source: Microorganisms. 2024 Oct 7;12(10):2025. doi: 10.3390/microorganisms12102025 (PMC11509576; doi:10.3390/microorganisms12102025)

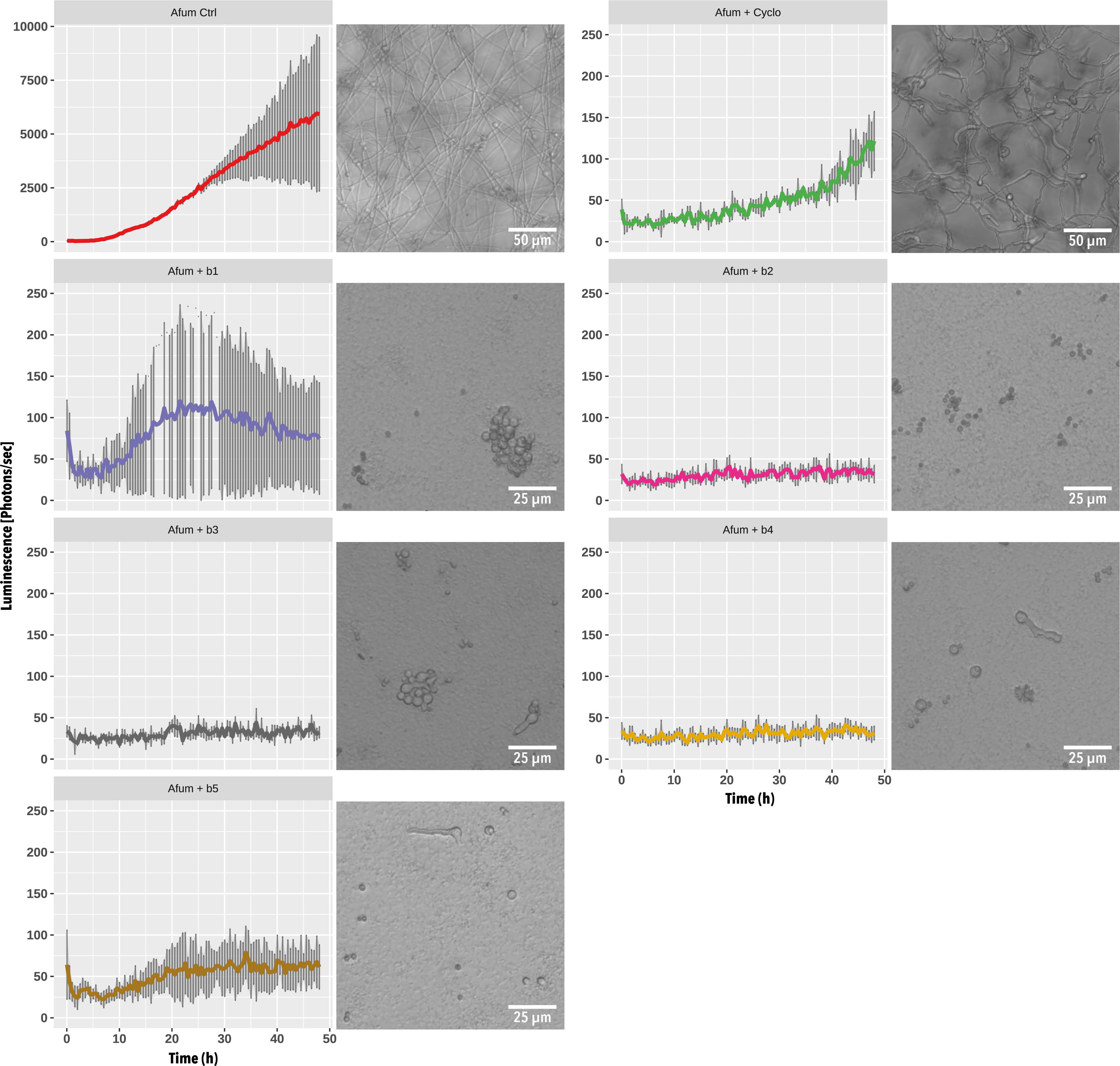

Supplement: Supplementary file 1 [file microorganisms-12-02025-s001.zip › Figure S1.png]

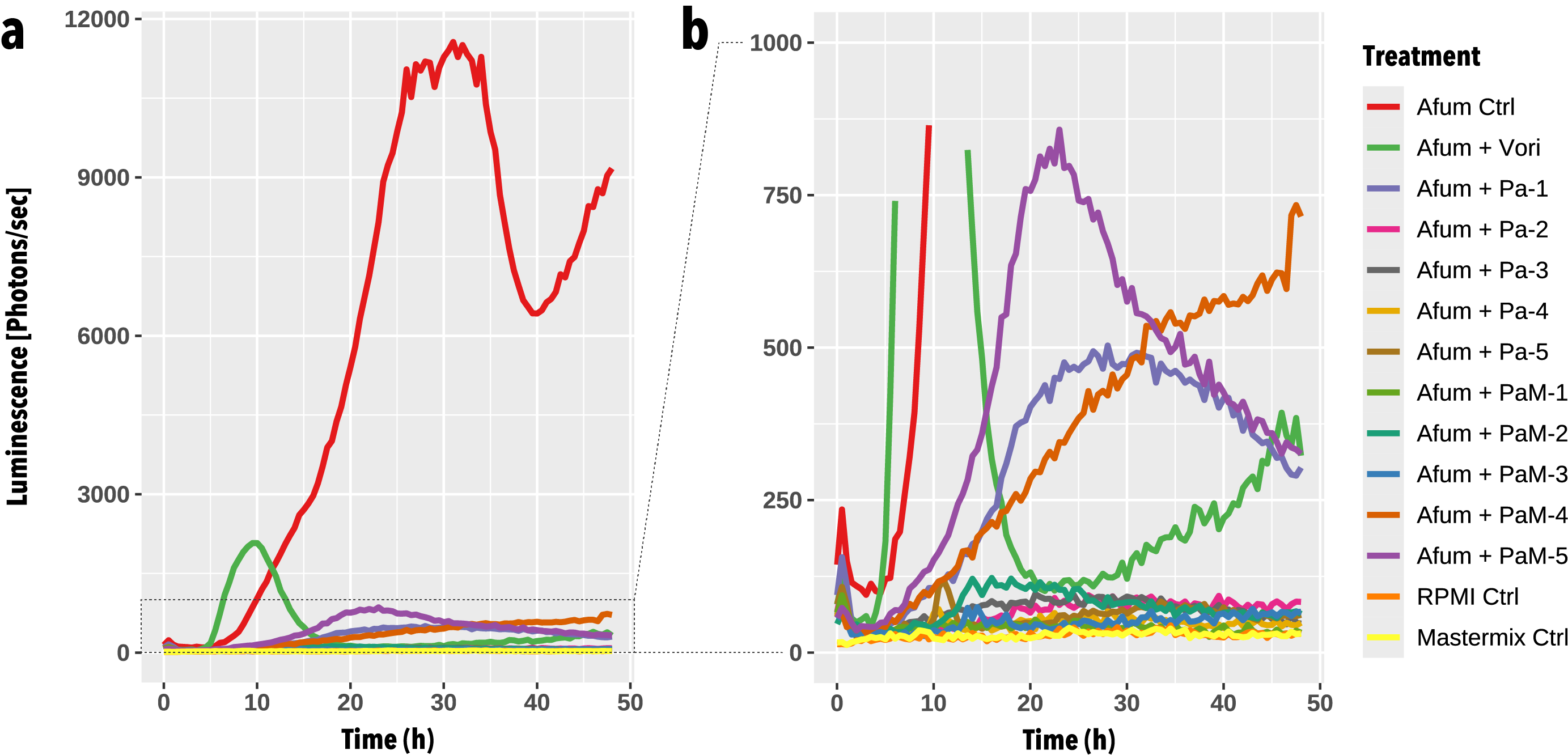

Supplement: Supplementary file 1 [file microorganisms-12-02025-s001.zip › Figure S2.png]

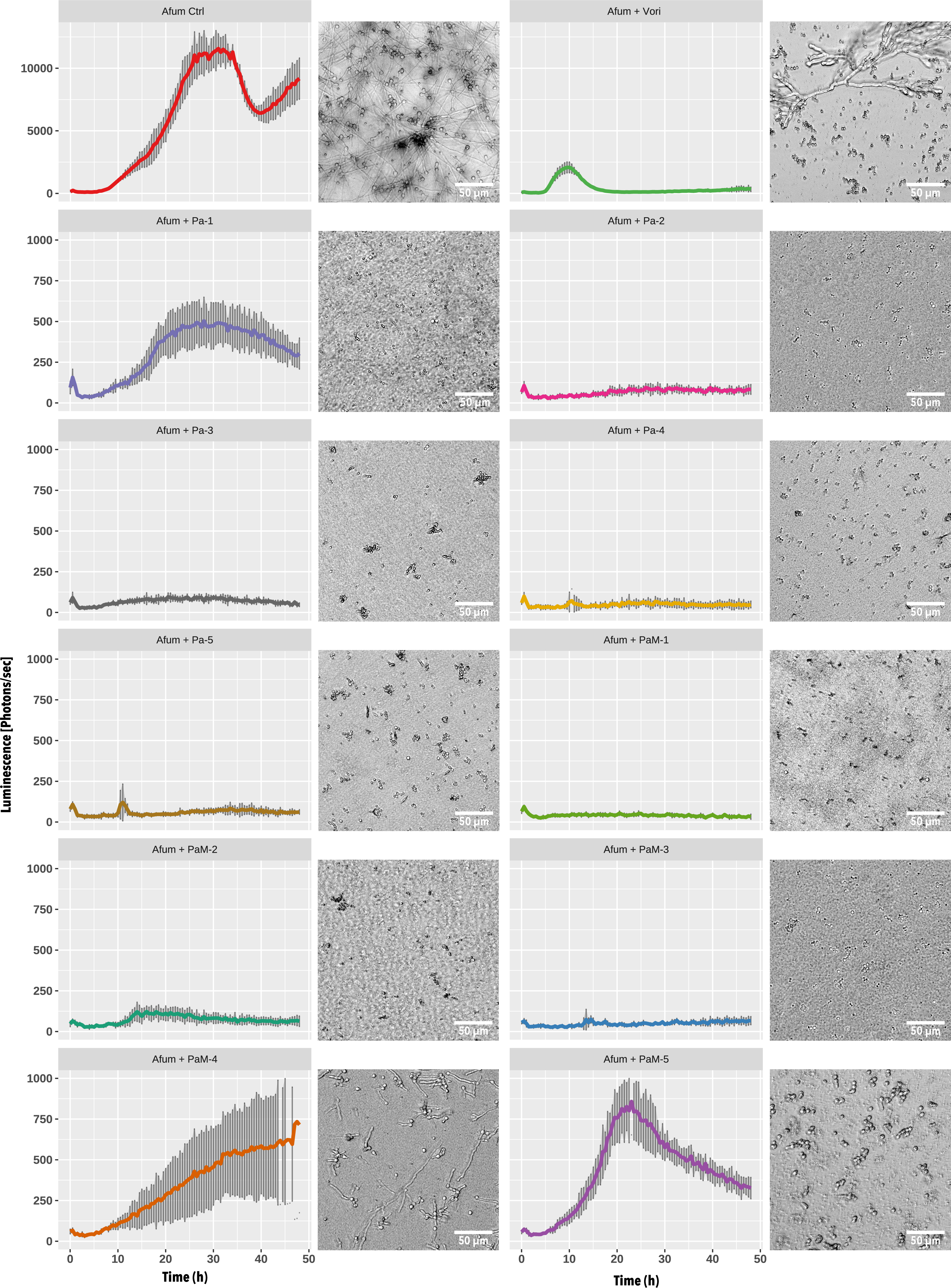

Supplement: Supplementary file 1 [file microorganisms-12-02025-s001.zip › Figure S3.png]

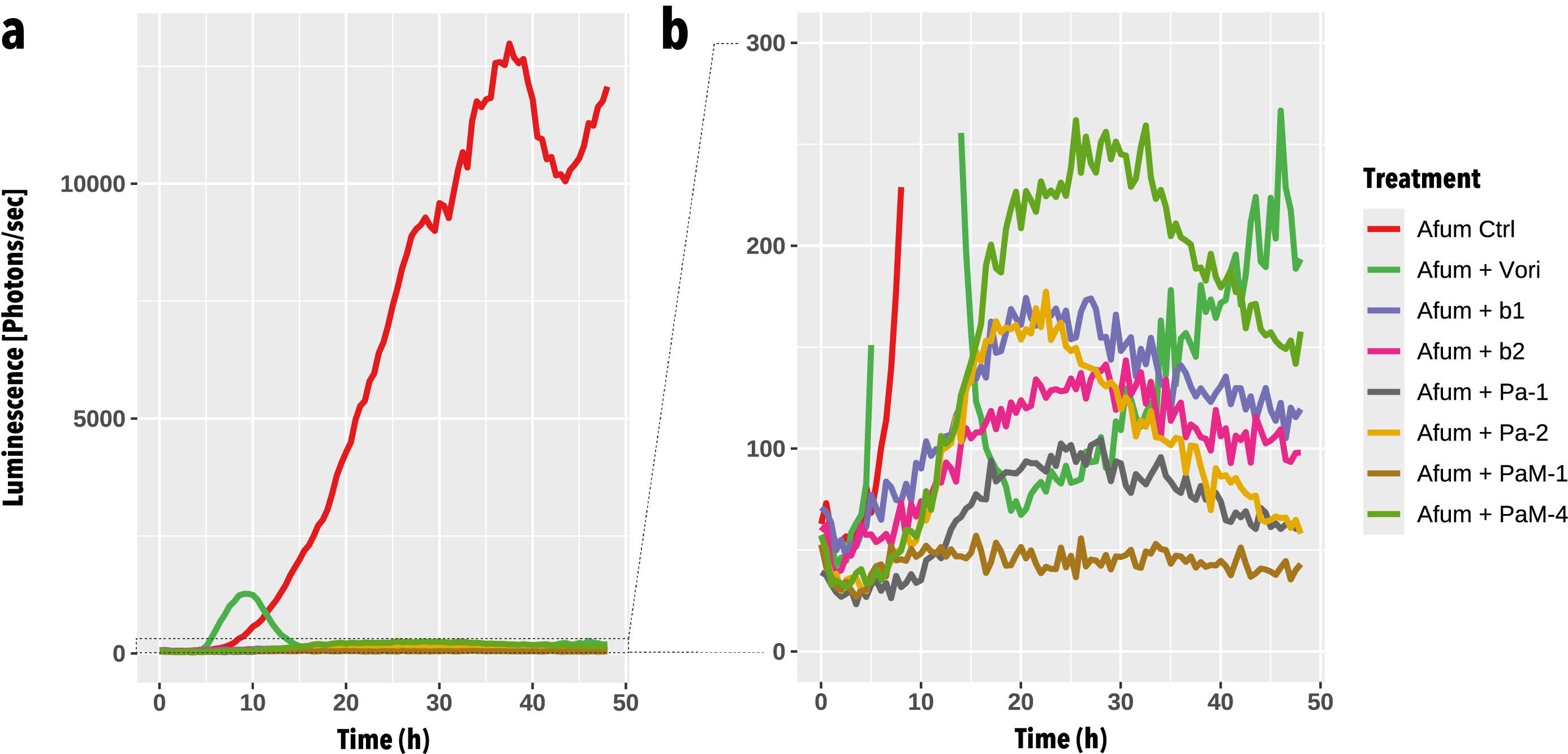

Supplement: Supplementary file 1 [file microorganisms-12-02025-s001.zip › Figure S4.png]

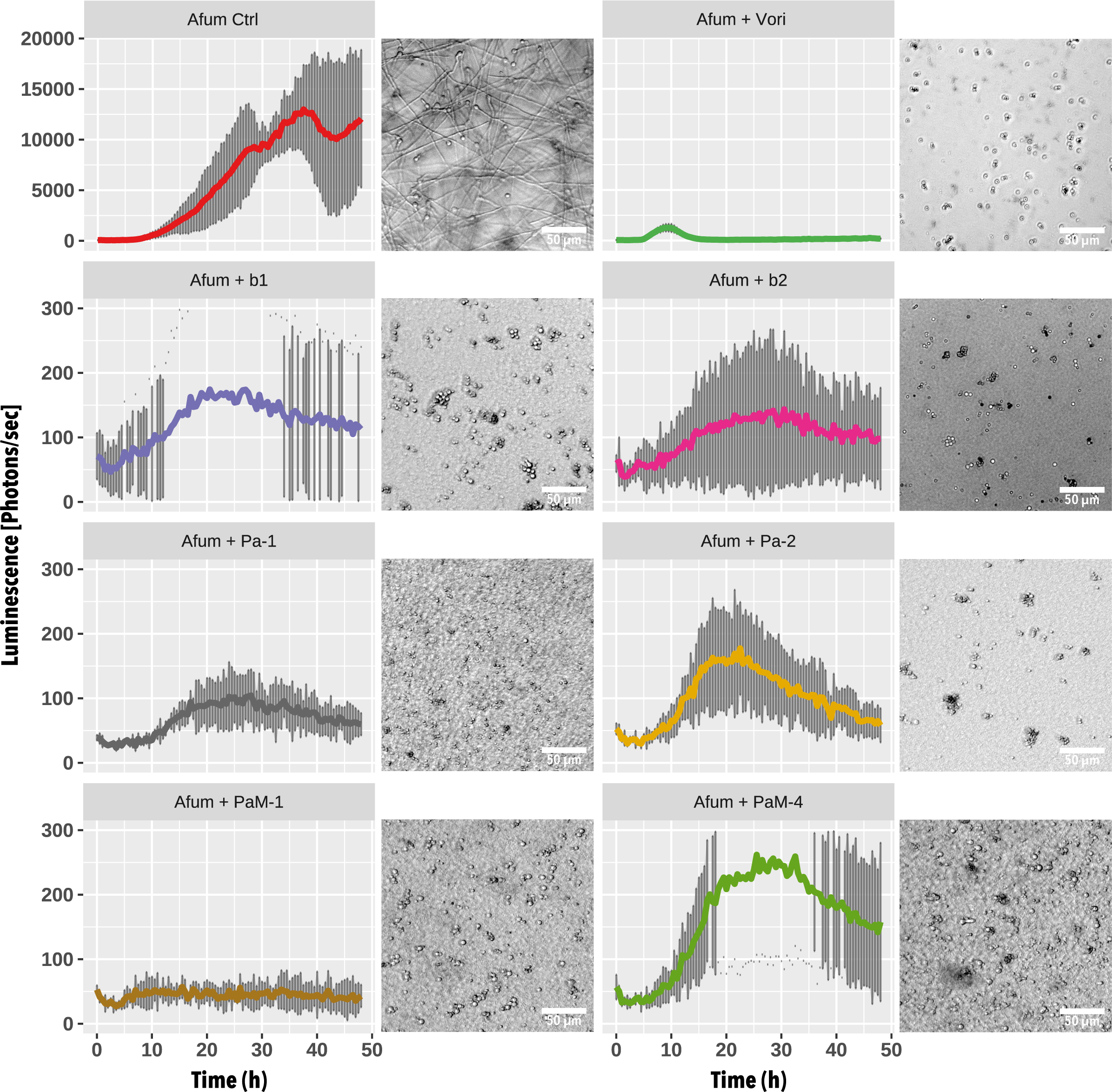

Supplement: Supplementary file 1 [file microorganisms-12-02025-s001.zip › Figure S5.png]

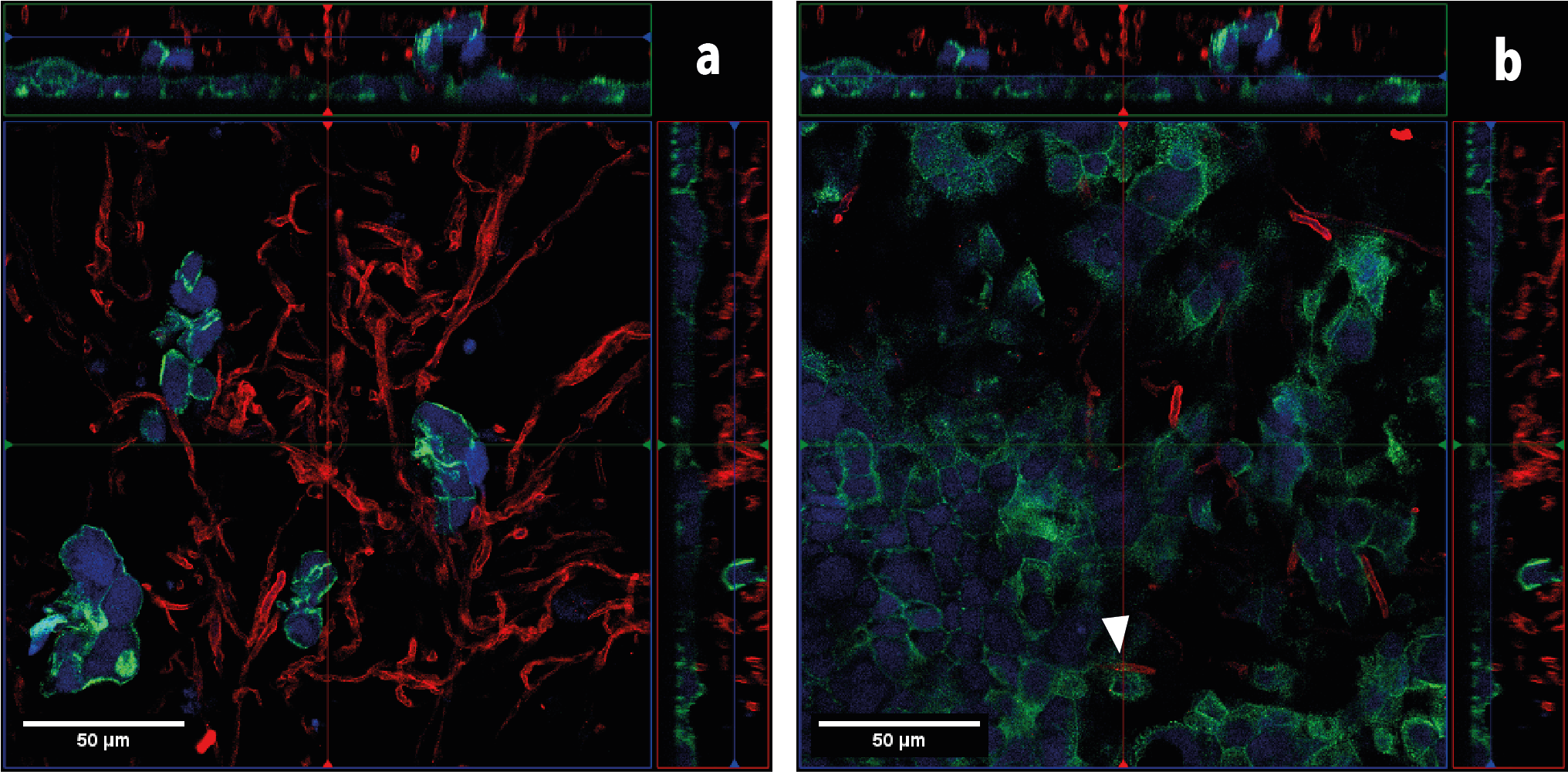

Supplement: Supplementary file 1 [file microorganisms-12-02025-s001.zip › Figure S6.png]
